# Neutrophil- and Endothelial Cell-Derived Extracellular Microvesicles Are Promising Putative Biomarkers for Breast Cancer Diagnosis

**DOI:** 10.3390/biomedicines13030587

**Published:** 2025-02-27

**Authors:** Thayse Batista Moreira, Marina Malheiros Araújo Silvestrini, Ana Luiza de Freitas Magalhães Gomes, Kerstin Kapp Rangel, Álvaro Percínio Costa, Matheus Souza Gomes, Laurence Rodrigues do Amaral, Olindo Assis Martins-Filho, Paulo Guilherme de Oliveira Salles, Letícia Conceição Braga, Andréa Teixeira-Carvalho

**Affiliations:** 1 Grupo Integrado de Pesquisas em Biomarcadores, Instituto René Rachou-Fiocruz, Belo Horizonte 30190-002, Brazil; thaysebmoreira1@gmail.com (T.B.M.); msilvestrini@aluno.fiocruz.br (M.M.A.S.); olindo.filho@fiocruz.br (O.A.M.-F.); 2 Laboratório de Pesquisa Translacional em Oncologia, Instituto de Ensino, Pesquisa e Inovação, Instituto Mário Penna, Belo Horizonte 30380-420, Brazil; sallespgo@gmail.com; 3 Hospital Luxemburgo, Instituto Mário Penna, Belo Horizonte 30380-420, Brazil; analuizafmg@gmail.com (A.L.d.F.M.G.); kerstinkapprangel@hotmail.com (K.K.R.); alvaropercinio@gmail.com (Á.P.C.); 4 Laboratório de Bioinformática e Análise Molecular, Universidade Federal de Uberlândia (UFU), Campus Patos de Minas, Patos de Minas 38701-002, Brazil; souzagomes.matheus@gmail.com (M.S.G.); laurence.amaral@gmail.com (L.R.d.A.); 5 Laboratório de Anatomia Patológica, Hospital Luxemburgo, Instituto Mário Penna, Belo Horizonte 30380-420, Brazil

**Keywords:** breast cancer, liquid biopsy, extracellular microvesicles, flow cytometry, biomarkers

## Abstract

**Introduction:** Breast cancer (BC) is a disease that affects about 2.2 million people worldwide. The prognosis and treatment of these patients depend on clinical and histopathologic staging, in which more aggressive cancers need a less conservative therapeutic approach. Previous studies showed that patients with BC have an increased frequency of systemic microvesicles (MVs) that are associated with invasion, progression, and metastasis, which can be used in liquid biopsy to predict the therapeutic response in individualized treatment. **Objective:** This study proposes the development of a minimally invasive BC diagnostic panel and follow-up biomarkers as a complementary method to screen patients. **Methods:** The quantification of circulating MVs in 48 healthy women and 100 BC patients who attended the Mário Penna Institute between 2019 and 2022 was performed by flow cytometry. In addition, the MVs of BC patients were analyzed before treatment and 6, 12, and 24 months post-treatment. Machine learning approaches were employed to determine the performance of MVs to identify BC and to propose BC classifier algorithms. **Results:** Patients with BC had more neutrophil- and endothelial cell-derived MVs than controls before treatment. After treatment, all MV populations were decreased compared to pre-treatment, but leukocyte- and erythrocyte-derived MVs were increased at 12 months after treatment, before decreasing again at 24 months. **Conclusions:** Performance analyses and machine learning approaches pointed out that MVs from neutrophils and endothelial cells are the best candidates for BC diagnostic biomarkers. Neutrophil- and endothelial cell-derived MVs are putative candidates for BC biomarkers to be employed as screening tests for BC diagnosis.

## 1. Introduction

According to the World Health Organization, breast cancer (BC) is a malignant neoplasm that affected about 2.2 million people in 2020 [1]. In Brazil, BC is the most prevalent cancer affecting the female population, and around 74 thousand new cases were expected in 2023 [2]. The prognosis and treatment of patients depend on clinical and anatomopathological staging, which is performed by core biopsy, an invasive and painful procedure. Recently, the molecular characteristics of tumors have been considered for clinical evaluation and treatment response [3,4]. Liquid biopsy has been developed using body fluids such as blood to obtain more accurate information and lower invasiveness [5,6]. Several molecules have been tested for this purpose, such as free nucleic acids, tumor stem cells, and, more recently, extracellular microvesicles (MVs) [7,8].

MVs are extracellular vesicles released by all cells in physiological and pathological conditions [9]. In this sense, in breast cancer, high concentrations of MVs are found [10]. These MVs carry markers capable of identifying the cellular origin and molecules that can act as modulators of the immune response in tumor development and progression locally and at a distance, promoting the formation of new tumor niches [8,11,12,13,14,15]. Some studies report that, during tumor progression, there is an increased number of MVs released by immune cells into the tumor microenvironment, and these changes can be identified in the patient’s blood through liquid biopsy [16,17,18]. In addition, all cell types that form the tumor microenvironment (TEM) are capable of secreting MVs, and the type of stimulus will define their cargo [19]. Important processes for the development and progression of the tumor are impacted by the extracellular vesicles, such as angiogenesis which is influenced by the MVs from endothelial cells, platelets, and erythrocytes [20].

Considering the importance of BC for public health and the need to develop noninvasive new diagnostic tools for BC, this study evaluated the potential use of circulating MVs in liquid biopsy for a less invasive and accurate diagnosis to screen BC and as a monitoring tool for affected women.

## 2. Population, Materials and Methods

### 2.1. Patient Cohort

This prospective observational study was conducted between 2019 and 2022 at the Mário Penna Institute, Belo Horizonte, Minas Gerais State, Brazil. The case group included 100 patients with locally advanced breast cancer confirmed by histological exam, with the indication of chemotherapy and radiotherapy. The patients did not present other neoplasms. The control group comprised 48 healthy women with mammograms without abnormal pathological findings. Clinicopathological data were obtained from medical records, and a questionnaire was applied to the control group. Informed consent was obtained from all included patients. The study was submitted and approved by the Ethics Committee at the Mario Pena Institute (CAEE 82703418.8.0000.5121).

### 2.2. Blood Collections 

Peripheral blood samples were collected before the start of treatment and at 6, 12, and 24 months after the end of chemotherapy and radiotherapy sections. The samples were collected using vacuum tubes containing 3.2% sodium citrate (Vacutainer Blood Collection Tube, BD Medical, Franklin Lakes, NJ, USA). The samples were centrifuged at 600× *g* for 15 min, at room temperature to obtain platelet-poor plasma (PPP). Aliquots of 1 mL were stored in a freezer at −80 °C until use.

### 2.3. Immunophenotypic Analysis of MVs by Flow Cytometry

The PPP samples were thawed in a 37 °C water bath, diluted in a citrate buffer solution containing heparin (1 μg/mL), and centrifuged at 1500× *g* for 90 min at 15 °C. The sediment containing the MVs was resuspended in commercial Annexin V buffer (25 mM CaCl_2_ solution in 140 mM NaCl and 10 mM HEPES, pH 7.4; BD Biosciences, San Diego, CA, USA). To determine the cellular origin of the MVs, 100 µL of the suspension was transferred to tubes containing 2 µL of monoclonal antibodies conjugated to different fluorochromes, namely, APC-CD45 (clone HI30), PE-CD66b (clone B1.1), PE-CD16 (clone 3G8), PE-CD51/CD61 (clone 23C6), PE-Cy5.5-CD235a (clone GA-R2/HIR2), PE-CD3 (clone HIT3a), PerCP-CD41a (clone HIP8), PerCP-CD14 (clone MEM-15), PE-HER2 (clone Neu 24.7), PE-Cy5.5-HSP27 (clone 155), and FITC-Annexin V (clone DX2), followed by incubation for 30 min in the dark, at room temperature.

In total, 1,000,000 events were acquired with at least 130,000 events within the annexin V-specific region using the flow cytometer Cytoflex S (Beckman-Coulter, Brea, CA, USA). Calibration microbeads (Gigamix beads, Stago Co, Marseille, France) with a 100–900 nm range were used to select the size range of MVs analyzed (Figures S1 and S2).

### 2.4. Statistical Analysis

Statistical analyses were performed using GraphPad Prism v9.0^®^ software (San Diego, CA, USA). To evaluate the data distribution, the Kolmogorov–Smirnoff test was performed. For data considered non-parametric, in the comparison of two groups, the Mann–Whitney test was performed, and in the comparison of three groups, the Kruskal–Wallis test was performed followed by Dunn’s post-test, with a confidence interval of 95%. Signature analyses were performed based on the global median of the groups.

Performance tests were carried out using the MedCalc software version 7.3.0 (Ostend, Belgium, URL https://www.medcalc.org/, accessed on 1 March 2023) to determine the cut-off value, sensitivity, specificity, and likelihood ratio. The area under the curve (AUC) was determined by GraphPad Prism v9.0^®^ software. For all analyses, values of *p* < 0.05 were considered statistically significant.

### 2.5. Development and Training of BC Classifier Algorithms

Decision trees were generated to classify the attributes from MVs and clinical data of BC patients’ datasets using the J48 method and employing the Waikato Environment for Knowledge Analysis (WEKA) software, version 3.6.11 (University of Waikato, Hamilton, New Zealand). The leave-one-out cross-validation (LOOCV) method was applied to validate the model’s accuracy and robustness and to generalize the decision tree results of a given statistical analysis of an independent dataset.

## 3. Results

### 3.1. Clinical Characteristics of the Cohort

This study evaluated 100 women diagnosed with breast cancer (cases) and 48 healthy volunteers with mammography without pathological findings (control). The clinical characteristics of the women are summarized in Table 1.

The volunteers of the BC group had an age > 40 years old, with 60% receiving adjuvant treatment, 42% presenting clinical staging II, and 64% presenting histological grade II. Inflammatory infiltrate was present in 41% of tumors. Estrogen receptors were present in 76%, progesterone receptors in 62%, and HER2 in 46% of the evaluated tumors. Patients with metastasis before chemotherapy treatment represented 35% of the BC group.

### 3.2. Microvesicle Profile Before Treatment

**Figure 1:**
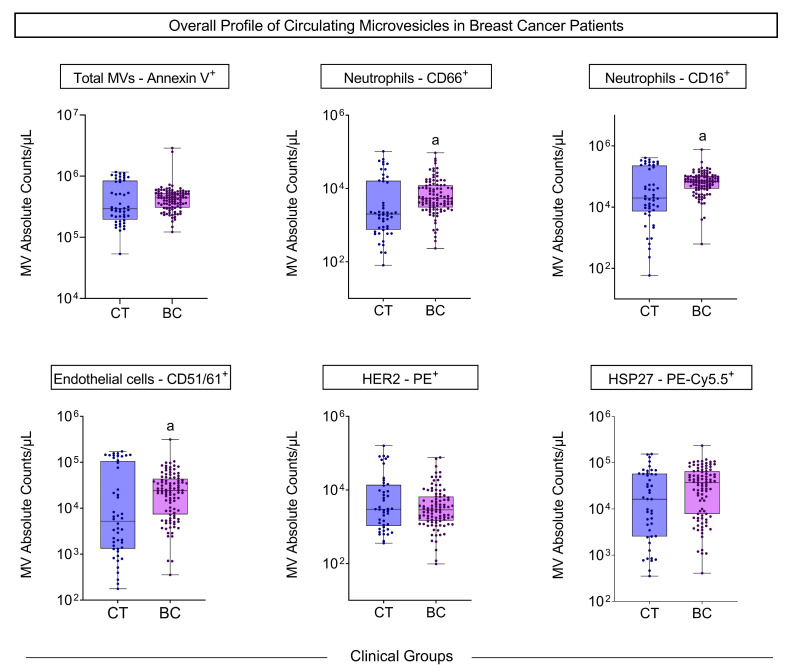
Overall profile of circulating microvesicles in the control group (CTRL, *n* = 48) and the case group (BC, *n* = 100). Boxes represent the median value and interquartile ranges of the absolute number of MVs in each group. Significant differences between the case and control groups are represented by the letter “a”.

The MV quantification showed a significant increase in circulating neutrophil- and endothelial cell-derived MVs from BC patients compared to the control group before starting chemotherapy and radiotherapy (Figure 1).

**Figure 2:**
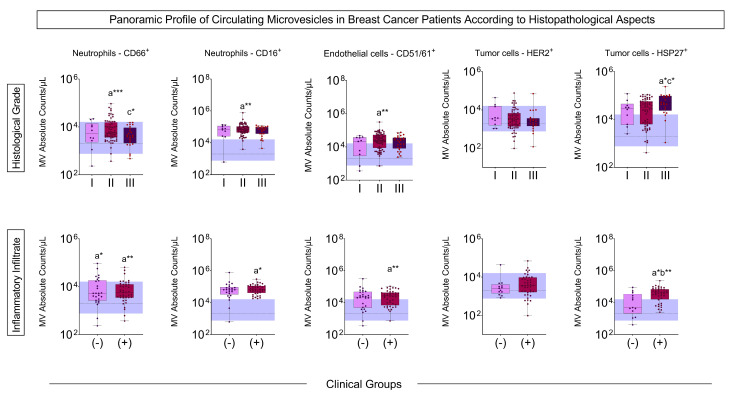
Panoramic profile of MVs according to histological grade and inflammatory infiltrate. Boxes represent the median value and interquartile ranges of the absolute number of MVs in each group. The dotted line represents the median and the blue band represents the interquartile ranges of the control group (*n* = 48). The letter ‘a’ indicates the differences compared to the control group, ‘b’ represents the differences compared to the group of patients without inflammatory infiltrate in the tumor tissue, and ‘c’ shows the differences in relation to the group of patients with histological grade II tumors. * *p* < 0.05, ** *p* < 0.01, *** *p* < 0.001.

The BC patients with the most advanced histological grade had an increased number of MVs of neutrophils, endothelial cells, and HSP27^+^ tumor cells compared to the control group and less aggressive tumor grades, as did the patients with the presence of inflammatory infiltrate when compared to control and patients without inflammatory infiltrate (Figure 2).

**Figure 3:**
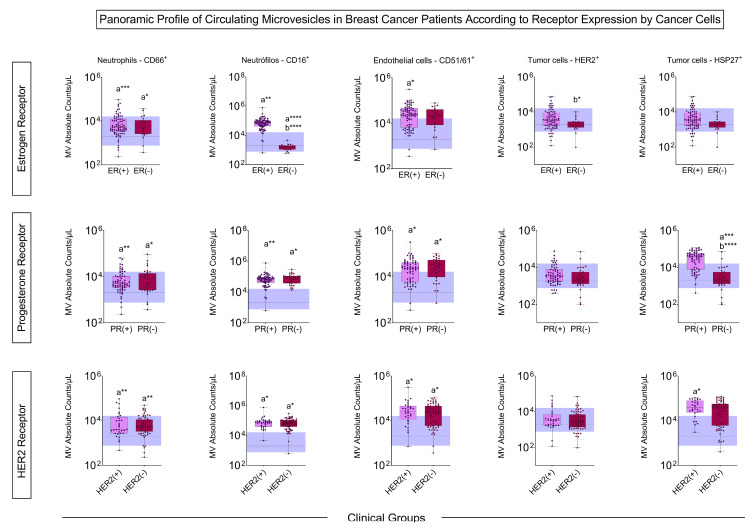
Quantification of MVs according to estrogen, progesterone, and HER2 receptors. Boxes represent the median value and interquartile ranges of the absolute number of MVs in each group. The dotted line represents the median and the blue band represents the interquartile ranges of the control group (*n* = 48). The letter ‘a’ indicates the differences compared to the control group, and ‘b’ represents the differences compared to the group of patients who present estrogen receptor (ER^+^) and Progesterone Receptor (PR^+^). The differences relative to the control group are illustrated by the letter “a”. * *p* < 0.05, ** *p* < 0.01, *** *p* < 0.001.

The patients with tumors expressing estrogen receptors had a more significant number of MVs from neutrophils and endothelial cells than the control, and those who did not express these receptors had an increase in MVs derived from CD66^+^ neutrophils compared to the control, and a decrease in MVs from CD16^+^ and HER2^+^ neutrophils compared to ER^+^. The profile of progesterone receptor-positive and -negative groups showed more MVs of neutrophils, endothelial cells, and HSP27^+^ tumor cells compared to the control. The same results were obtained concerning the HER2 receptor, except for HSP27^+^, which was only increased in patients who were HER2^+^ (Figure 3).

**Figure 4:**
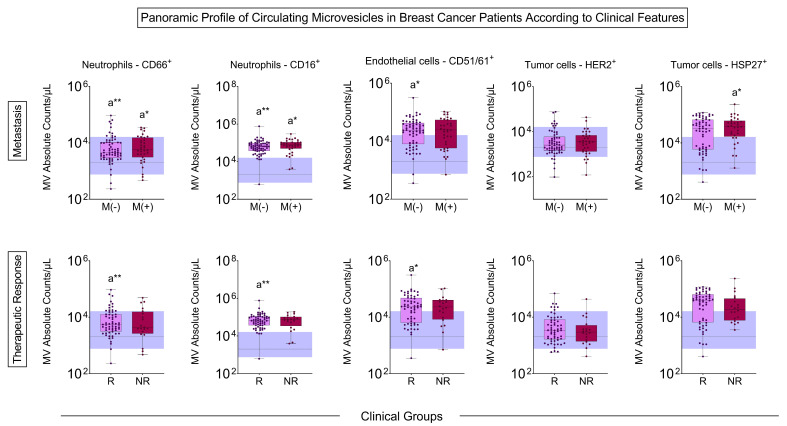
Panoramic profile of MVs according to clinical features before starting treatment. The profile of microvesicles was evaluated according to the presence (M(+), *n* = 36) or absence (M(−), *n* = 63) of metastasis and treatment responder (R, *n* = 60) and non-responder (NR, *n* = 20). Boxes represent the median value and interquartile ranges of the total number of MVs in each group. The dotted line represents the median and the blue band represents the interquartile ranges of the control group (*n* = 48). The differences relative to the control group are illustrated by the letter “a”. * *p* < 0.05, ** *p* < 0.01.

In the MV profile according to pre-treatment metastasis, there was an increase in neutrophil MVs in both BC patients with and without metastasis. In contrast, endothelial cell MVs increased in patients without metastasis, and HSP27^+^ tumor cells increased in metastatic BC patients. Patients who responded to treatment had a higher number of neutrophil- and endothelial cell-derived MVs than controls (Figure 4).

### 3.3. Microvesicle Profile After Treatment

**Figure 5:**
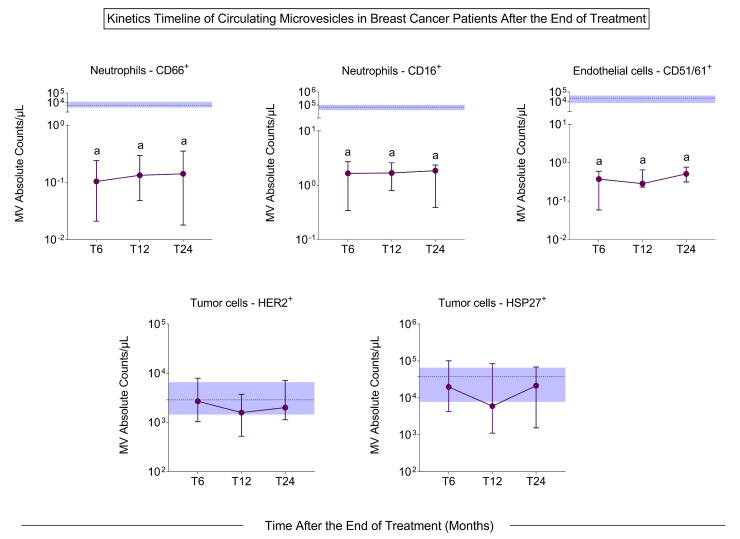
Quantification of circulating MVs in BC patients after 6, 12, and 24 months after the end of chemotherapy and radiotherapy. The dotted line represents the median value of the total number of MVs in each group and the blue band represents the interquartile ranges of the pre-treatment group (*n* = 100). The significant differences at times T6, T12, and T24 in relation to T0 are represented by the letter “a”. T6: six months post-treatment (*n* = 11); T12: 12 months post-treatment (*n* = 13); T24: 24 months post-treatment (*n* = 11).

The profile of the MVs was evaluated 6, 12, and 24 months after the end of chemotherapy and radiotherapy. It was observed that the number of MVs decreased significantly at 6, 12, and 24 months after treatment in all evaluated populations compared to pre-treatment (Figure 5), except for MVs related to tumor cells (HER2^+^ and HSP27^+^) (Figure S3). 

### 3.4. Signature Profile of Circulating MVs in BC Patients

**Figure 6:**
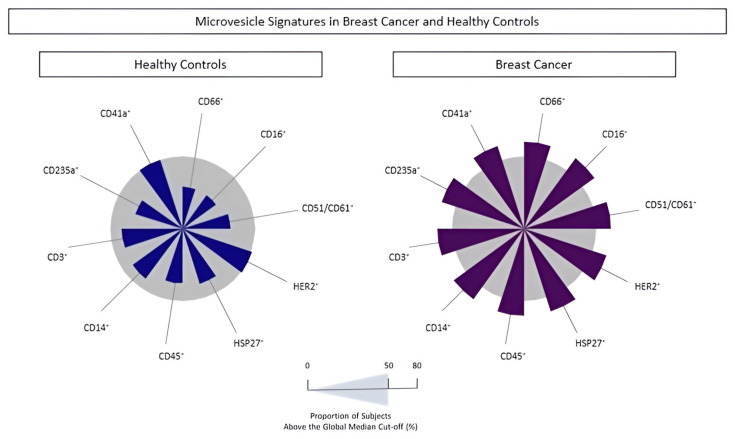
Microvesicle signature in breast cancer patients (*n* = 100) compared to healthy donors (*n* = 48). Radar graphs using the percentage of individuals above the global median were used as the cut-off point. CD45^+^: leukocytes; CD66^+^: neutrophils, CD51/CD61^+^: endothelial cells, CD235a^+^: erythrocytes, CD3^+^: T lymphocytes, CD41a^+^: platelets, CD16^+^: neutrophils, CD14^+^: monocytes, HER2^+^ and HSP27^+^: tumor cells.

The populations of MVs were analyzed to identify the signature profile using the global median values for each phenotypic feature. BC patients had increased circulating MVs from all cell subsets, except for platelet- and HER2^+^ tumor cell-derived MVs compared to controls (Figure 6). The proportion of individuals with MVs above the global median was 60% and 58% for neutrophil- and endothelial cell-derived MVs, respectively. These findings reinforce the idea that both MVs can be used as possible biomarkers for BC diagnosis.

### 3.5. BC Biomarkers Performance Index to Identify Subgroups of Patients with Cancer

**Figure 7:**
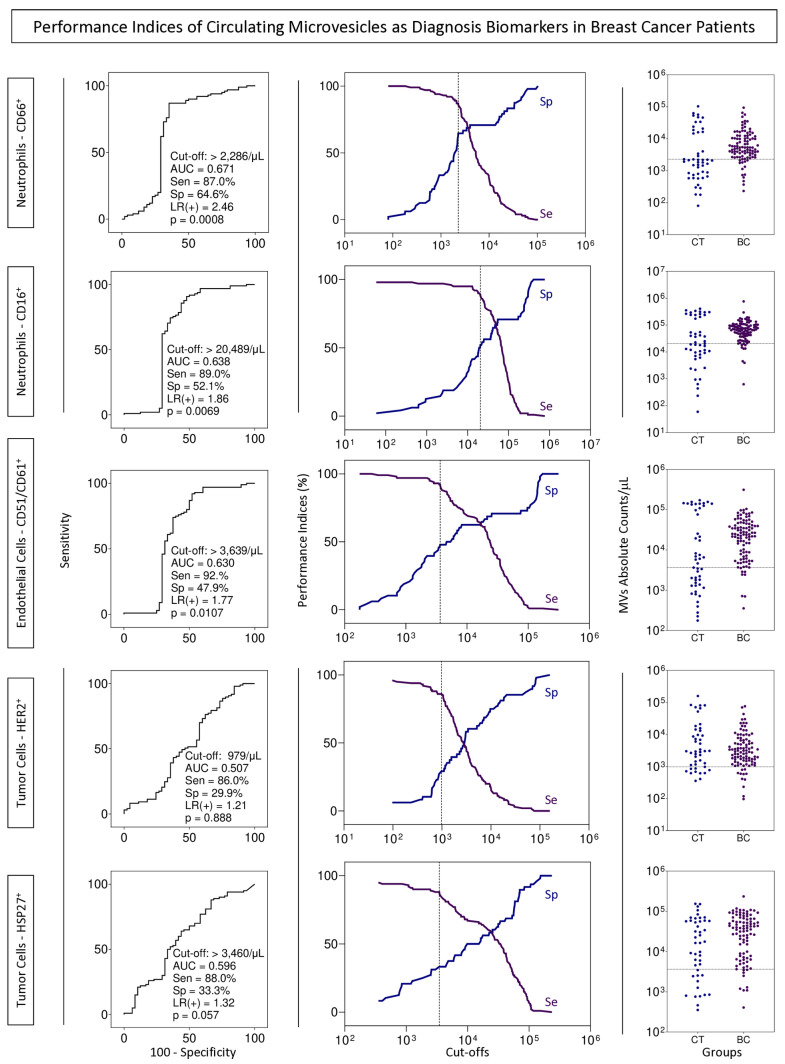
Performance indices of biomarkers in patients with breast cancer. Performance tests were carried out considering the diagnostic potential of microvesicles from CD66^+^ neutrophils, CD16^+^ neutrophils, CD51/CD61^+^ endothelial cells, HER2^+^ tumor cells, and HSP27^+^ tumor cells. The graphs on the left show the ROC curves containing the respective cut-off values, area under the curve (AUC), sensitivity (Se), specificity (Sp), positive likelihood ratio [LR(+)], and *p* value; the middle graphs show the TG-ROC curves, representing sensitivity (Se) and specificity (Sp) values, according to different cut-off points; The graphs on the right show the dispersion of the values of the MVs.

Performance tests for using MVs from neutrophils and endothelial cells as possible BC biomarkers by the liquid biopsy approach were executed to define the cut-off value, AUC, sensitivity, specificity, and likelihood ratio (Figure 7).

### 3.6. Proposal of Decision Tree Algorithm to Classify Patients with BC

**Figure 8:**
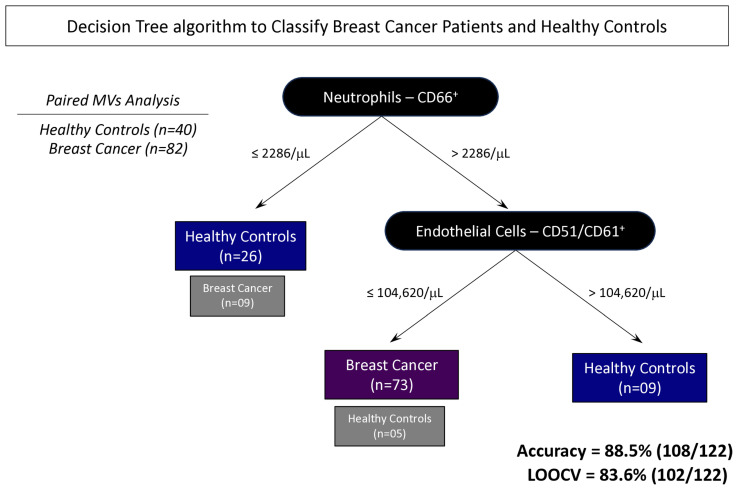
Decision tree algorithms were constructed to classify subgroups of patients with and without BC using neutrophil- and endothelial cell-derived MVs for the prediction of BC diagnosis model as a liquid biopsy strategy. For method validation, leave-one-out cross-validation (LOOCV) was used. Global accuracy and LOOCV were 88.5% [108/122] and 83.6% [102/122], respectively.

Based on the overall global accuracy observed for the neutrophil- and endothelial cell-derived MVs evaluated in BC patients on the above analysis, decision tree algorithms were developed to obtain a mathematical model to categorize subgroups of patients with and without BC. In this study, the classifier displayed a high global accuracy (88.5% [108/122]) in identifying BC patients and those without this disease, with a cross-validation value by a leave-one-out strategy of 83.6% [102/122] (Figure 8).

## 4. Discussion

BC is a lethal disease that affects many women in the world. Early diagnosis is the main goal in the fight against BC to improve outcomes and survival rates [1]. The diagnosis of BC is currently made by imaging and anatomopathological examination, which needs core biopsy invasive and high-cost methods that restrict access to the general population, making it necessary to develop new tools capable of popularizing access [21].

The tumor microenvironment has a complex and diverse structure composed of tumor cells, immune and stromal cells, blood vessels, and extracellular matrix (ECM). One of the most studied forms of communication in this complex tumor microenvironment are MVs, which are structures that carry molecules capable of identifying their origin and are very sensitive to environmental changes [14].

Our study showed that patients with BC have more MVs of neutrophils and endothelial cells than controls in all situations considered prognostic, such as histological grade, inflammatory infiltrate, the presence of hormonal markers, and metastasis (Figure 1, Figure 2, Figure 3 and Figure 4). Neutrophil-derived MVs are known to be present in the tumor microenvironment, influenced by the activation state of immune cells, transporting cellular mediators, and affecting neutrophil chemotaxis [22]. Tumor-associated neutrophils (TANs) are cells in the tumor microenvironment and may have an anti-tumor or pro-tumor profile based on clinical–pathological characteristics. The primary function of TANs during tumor progression is to promote angiogenesis and invasion by releasing molecules, such as proteases and growth factors, modifying the ECM [23]. The local delivery of neutrophil-derived MVs leads to the recruitment of new neutrophils and other immune system cells, besides carrying cytokines that modulate adhesion to endothelial cells, promoting diapedesis [24]. Zhang et al. [25] showed that when tumor cell lines of human hepatoma (HepG2), human gastric cancer (HGC27), and human colon cancer (SW480) are treated with neutrophil-derived MVs, the levels of apoptosis proteins, as caspase-3 and caspase-7, increased. Instead, it was seen that these MVs had FasL, granzyme A, granzyme B, and perforin in their composition, suggesting their role in the anti-tumor apoptotic process.

Neutrophils hold significant importance in cancer biology, with the neutrophil-to-lymphocyte ratio (NLR) being recognized as a prognostic marker in breast cancer (BC). Alshamsan et al. [26] demonstrated that elevated NLR is associated with poorer clinical outcomes, suggesting its potential as an independent prognostic indicator for both pathological response to treatment and survival, regardless of histological grade or hormone receptor status. Furthermore, Schmidt et al. [27] demonstrated that the increased concentration of intratumoral CD66^+^ neutrophils is associated with tumor progression and long-term metastasis, both in the tumor and adjacent tissues, as well as in the lymph nodes. The present study aims to expand on these findings by investigating the role of circulating microvesicles, given their high concentration in blood, as novel biomarkers in the prognostic landscape of BC.

The increase in MVs derived from endothelial cells is involved in tumor progression by acting on angiogenesis, stimulating vessel growth, and facilitating tumor cell diapedesis [28]. These MVs appear to have a pro-angiogenic function through the activity of metalloproteinases (MMPs), such as MMP-2 and MMP-9, acting on the ECM composition and integrity and stimulating new vessel formation. On the other hand, they can increase the production of reactive oxygen species, inhibiting the formation of new vessels. This difference may be due to the concentration of MVs in the tumor microenvironment, which, at low concentrations, promote angiogenesis and, at high concentrations, inhibit this process [29,30]. Tumor-associated endothelial cells produce IL-3, a pro-inflammatory cytokine. Lombardo et al. [31] showed that endothelial cell-derived MVs, in an environment with high concentrations of IL-3, act as a paracrine mediator of angiogenesis, blocking it and preventing new vessel formation. In small-cell lung tumors, the patients with a higher number of circulating endothelial cell-derived MVs have less than one year of survival after diagnosis whereas those with fewer MVs have more than one year of survival [32]. These data highlight the potential of endothelial cell-derived MVs as a prognosis biomarker.

In addition to MVs from the immune system and stromal cells, the tumor microenvironment is rich in MVs from its tumor cells. These cells act in communication with (i) membrane proteins, carrying out drug efflux and promoting drug resistance; and (ii) genetic material, i.e., DNA and miRNAs that modulate gene expression and modify the environment. Due to this, MVs allow tumor growth and progression, and transport membrane-anchored cytokines and chemokines that can act in cell modulation and immune recruitment [33].

Our study showed increased HSP27^+^ MVs in tumors with a worse prognosis, such as those with histological grade III, the presence of inflammatory infiltrate, and pre-treatment metastasis. It has already been seen that patients with tumors with a higher histological grade (II–III) have more expression of HSP27, as well as patients who co-express HER2 [34]. HSP27 is a chaperone known to be overexpressed in BC, especially in aggressive tumors, acting on the migration and invasion of tumor cells, promoting metastasis [35]. In addition, the tumor microenvironment has high levels of soluble HSP27. This soluble protein interacts with immunological cells infiltrated in the tumor and may induce the macrophages’ differentiation into non-responsive/anergic T cells, losing tumoricidal activity [36]. In evaluating the HSP27 presence of MVs in patients with BC, Liebhardt et al. [37] demonstrated that women with BC have a higher number of MVs of CD66 neutrophils and HSP27^+^ tumor cells when compared to controls, and so do women with lymph node metastasis when compared to patients without metastasis, corroborating our study. Our data show the possibility of using HSP27^+^ MVs as a complementary tool in staging patients with BC, providing information about the tumor in the bloodstream as a liquid biopsy.

After the end of the treatment, we observed that the MVs originating from immune cells decreased significantly compared to pre-treatment, and the MVs of tumor origin (HER2- and HSP27-derived) continued at the same frequency in the blood (Figure 3). Differently from the literature, this study proposed the use of MVs in the follow-up of patients after treatment to recognize patterns that allow the identification of BC recurrence before it is possible in the imaging exam. This work showed a considerable reduction in all populations of MVs at all times evaluated. However, after 12 months, there was a change in the profile of MVs, suggesting an increase in MVs derived from leukocytes, CD66^+^ neutrophils, and erythrocytes and a decrease in MVs of T lymphocytes, endothelial cells, and tumor cells. In the literature, Sierko et al. [38] showed that after three months of chemoradiotherapy, there was a decrease in endothelial cell MVs in head and neck cancer, demonstrating the interference of treatment in the concentration of MVs, but the other timepoints were not analyzed. Studies have reported the prognostic potential of MVs, for example, the study by Liu et al. [39]. They showed patients with non-small-cell lung cancer and disease complete remission had fewer MVs six months after treatment, while patients with disease progression had a large number of MVs compared to pre-treatment. With the results obtained in this study, the importance of investigating the profile of MVs after treatment as a possible follow-up tool is clear.

The profile of MVs of neutrophils and endothelial cells stands out from the initial analyses, emphasizing their importance in the BC development process. In all situations evaluated, these MVs were highlighted in patients with BC, and related to clinical characteristics known for a worse prognosis, such as those with moderately differentiated tumors (Figure 2). Evaluating MVs based on these aspects is innovative and provides more accurate information on the influence of these structures on BC. The signature profile demonstrated that these MVs were important BC biomarkers, with the MVs of neutrophils and endothelial cells achieving values above the global median of 60% and 58% (Figure 6), respectively, and showing a significant increase in BC patients. Thus, we proposed these MVs as potential diagnostic biomarkers. Performance indices as biomarkers were made to evaluate their clinical use individually. Neutrophil-derived MVs showed a sensitivity of more than 87%, and endothelial cell MVs a sensitivity of 92% (Figure 7). In addition, we performed artificial intelligence analysis using MVs of neutrophils and endothelial cells associated with clinicopathological characteristics. The algorithms showed that MVs alone could identify patients with BC with a global accuracy of 83.6% and 83.6% in leave-one-out cross-validation (LOOCV), and other features were not necessary (Figure 8).

The authors acknowledge this study’s limitations, including its limited sample size, especially within specific histopathological subgroups, its unicentric design, and its reliance on convenience sampling. Additionally, microvesicle characterization was restricted to flow cytometry. Despite these constraints, this study’s primary strength lies in its potential to improve breast cancer monitoring, particularly for women at high risk. By leveraging circulating MVs, either alone or integrated with artificial intelligence, this approach could inform future health policies for BC. Early-stage diagnosis, which can lead to significantly increased survival rates and improved quality of life with fewer side effects, highlights the critical importance of this research direction [40,41].

Our study proposes the use of neutrophil- and endothelial cell-derived MVs as circulating biomarkers to help the screening and diagnosis of patients with BC as a less invasive and more accessible tool. The application of the proposed algorithm could be helpful in supporting health professionals in making an accurate diagnosis, allowing early treatment and increasing patient survival. From a future perspective, further studies and the validation of our results are needed to translate these data into clinical care.

## Figures and Tables

**Table 1:** Clinical and pathological characteristics of the cohort.

Characteristics	Control Group		Cancer Group	
	*n* (%)	*p*-Value	*n* (%)	*p*-Value
Total	48 (100)	-	100 (100)	-
Age in years, median (IQR)	58 (51–63)	-	53 (43–60)	-
Age range				
18–49	10 (21)	<0.0001	35 (35)	**0.003**
50–73	38 (79)		65 (65)	
Family history of breast, ovarian, prostate, and bowel cancers				
Absent	30 (63)	0.0833	59 (59)	0.071
Present	18 (37)		41 (41)	
Treatment				
Adjuvant	N/A	-	60 (60)	**0.045**
Neoadjuvant	N/A	-	40 (40)	
Clinical staging based on the TNM system				
I	N/A	-	23 (23)	**<0.001**
II	N/A	-	42 (42)	
III	N/A	-	26 (26)	
IV	N/A	-	9 (9)	
Histological grade				
I	N/A	-	10 (10)	**<0.001**
II	N/A	-	64 (64)	
III	N/A	-	25 (25)	
N/A	N/A	-	1 (1)	
Inflammatory infiltrate in the tumor stroma				
Absent	N/A	-	15 (15)	**<0.001**
Present	N/A	-	41 (41)	
N/A	N/A	-	44 (44)	
Estrogen receptor				
Negative	N/A	-	22 (22)	**<0.001**
Positive	N/A	-	76 (76)	
N/A	N/A	-	2 (2)	
Progesterone receptor				
Negative	N/A	-	35 (35)	**0.006**
Positive	N/A	-	62 (62)	
N/A	N/A	-	3 (3)	
HER2				
Negative	N/A	-	66 (66)	0.058
Positive	N/A	-	46 (46)	
N/A	N/A	-	2 (2)	
Metastasis before chemotherapy treatment				
Absent	N/A	-	65 (65)	**0.012**
Present	N/A	-	35 (35)	

IQR: interquartile range; TNM: Classification of Malignant Tumors; N/A: not applicable. Data distribution was evaluated by chi-square. Values of *p* < 0.05 (bold) were considered statistically significant.

## Data Availability

The data presented in this study are available on request from the corresponding author due to participant confidentiality.

## Supplementary Materials

The following supporting information can be downloaded at: https://www.mdpi.com/article/10.3390/biomedicines13030587/s1, Figure S1: Analysis of strategies for the phenotypic characterization of EVs using flow cytometry according to size range; Figure S2: Analysis of strategies for the phenotypic characterization of EVs using flow cytometry; Figure S3: Quantification of circulating MVs in BC patients after 6, 12, and 24 months after the end of chemotherapy and radiotherapy. The dotted line represents the median value of the total number of MVs in each group and the blue band represents the interquartile ranges of the pre-treatment group (*n* = 100). The significant differences at times T6, T12, and T24 in relation to T0 are represented by the letter “a”. T6: six months post-treatment (*n* = 11); T12: 12 months post-treatment (*n* = 13); T24: 24 months post-treatment (*n* = 11).
