# Supplementary material for: Neutrophil- and Endothelial Cell-Derived Extracellular Microvesicles Are Promising Putative Biomarkers for Breast Cancer Diagnosis
Source: Biomedicines. 2025 Feb 27;13(3):587. doi: 10.3390/biomedicines13030587 (PMC11940338; doi:10.3390/biomedicines13030587)
Supplement: Supplementary file 1 [file biomedicines-13-00587-s001.zip › biomedicines-3235287-supplementary.pdf]

Supplementary materials

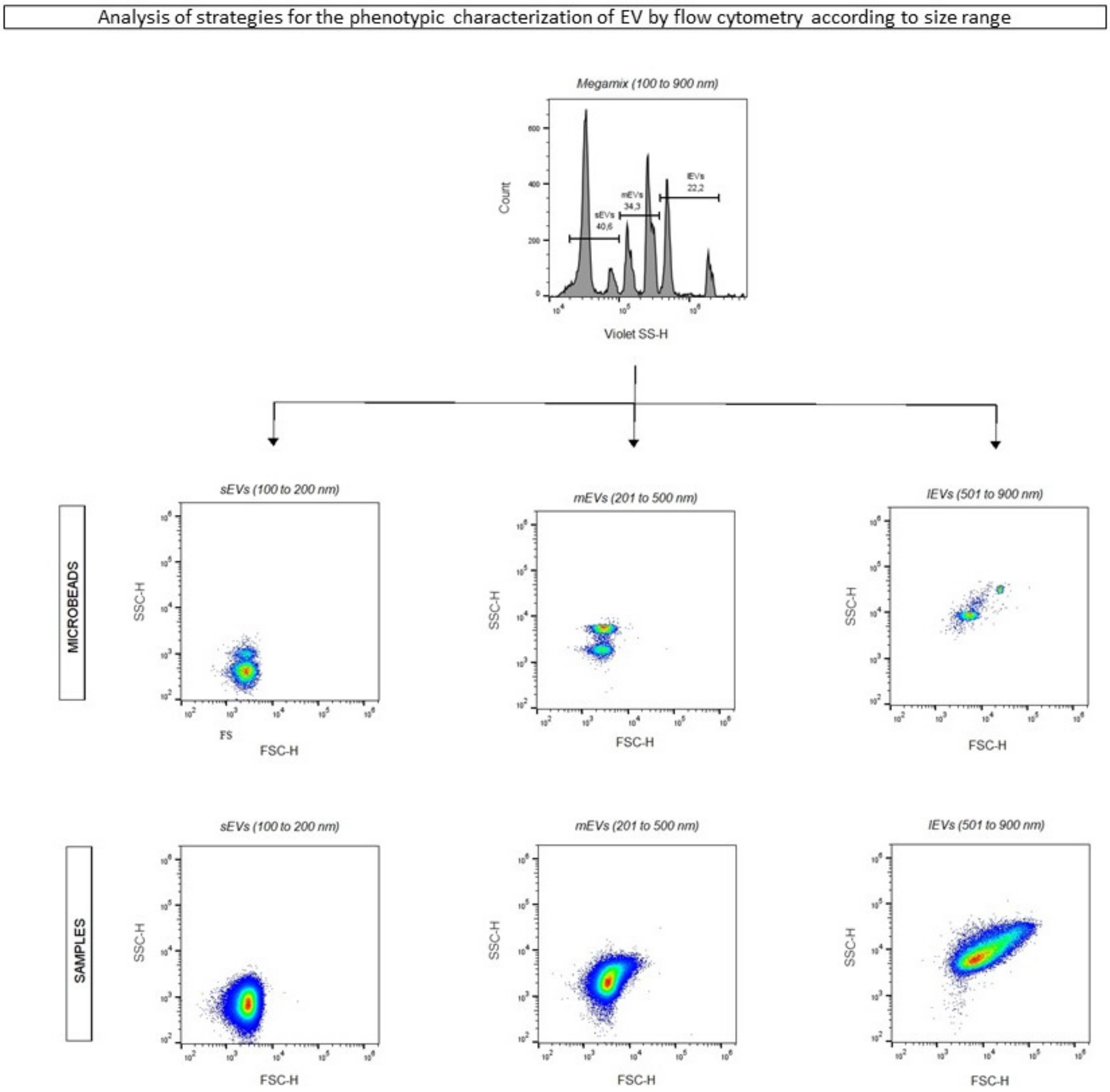

Figure S1: Analysis of strategies for the phenotypic characterization of EVs using flow cytometry according to size range.

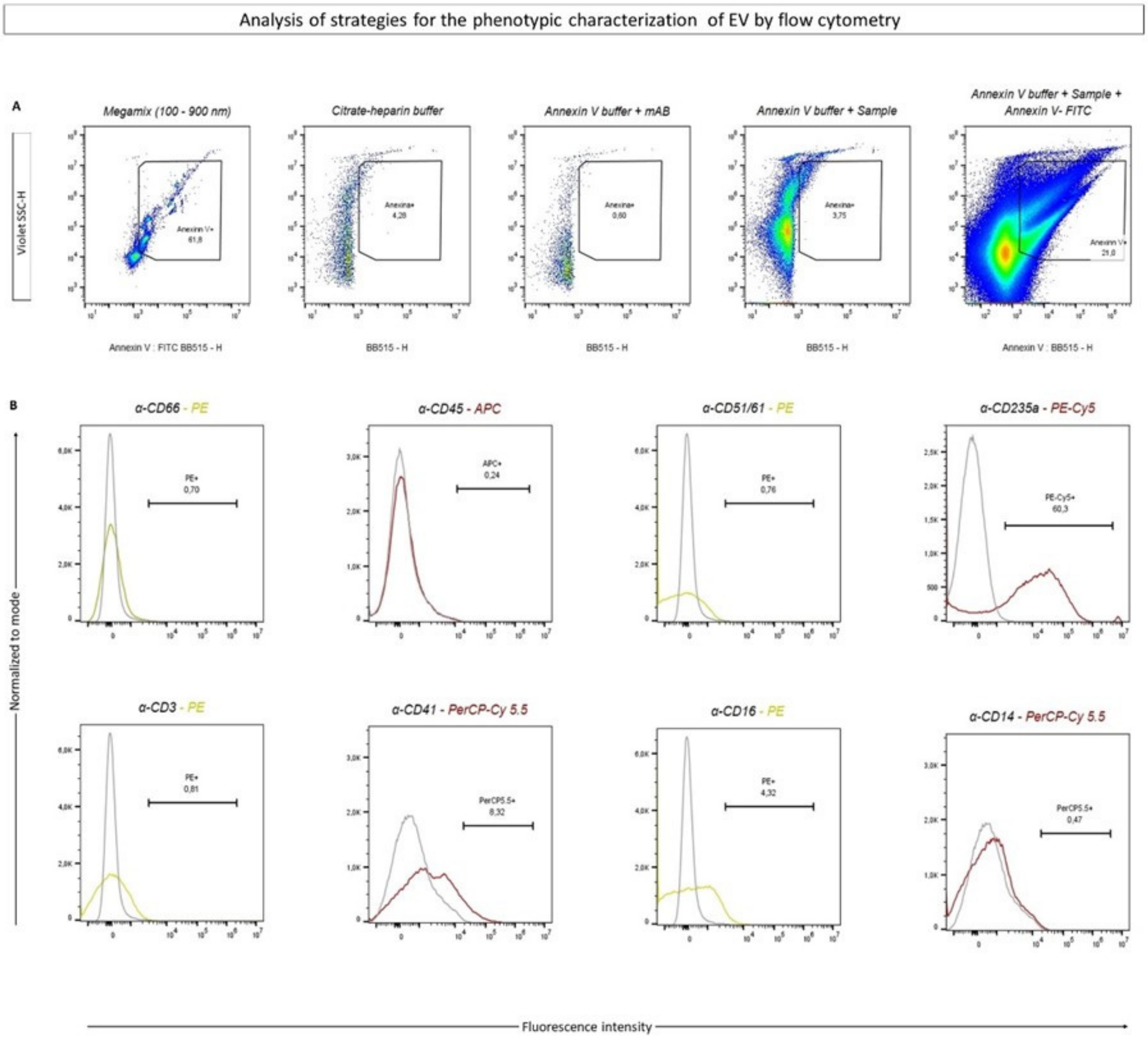

Figure S2: Analysis of strategies for the phenotypic characterization of EVs using flow cytometry.

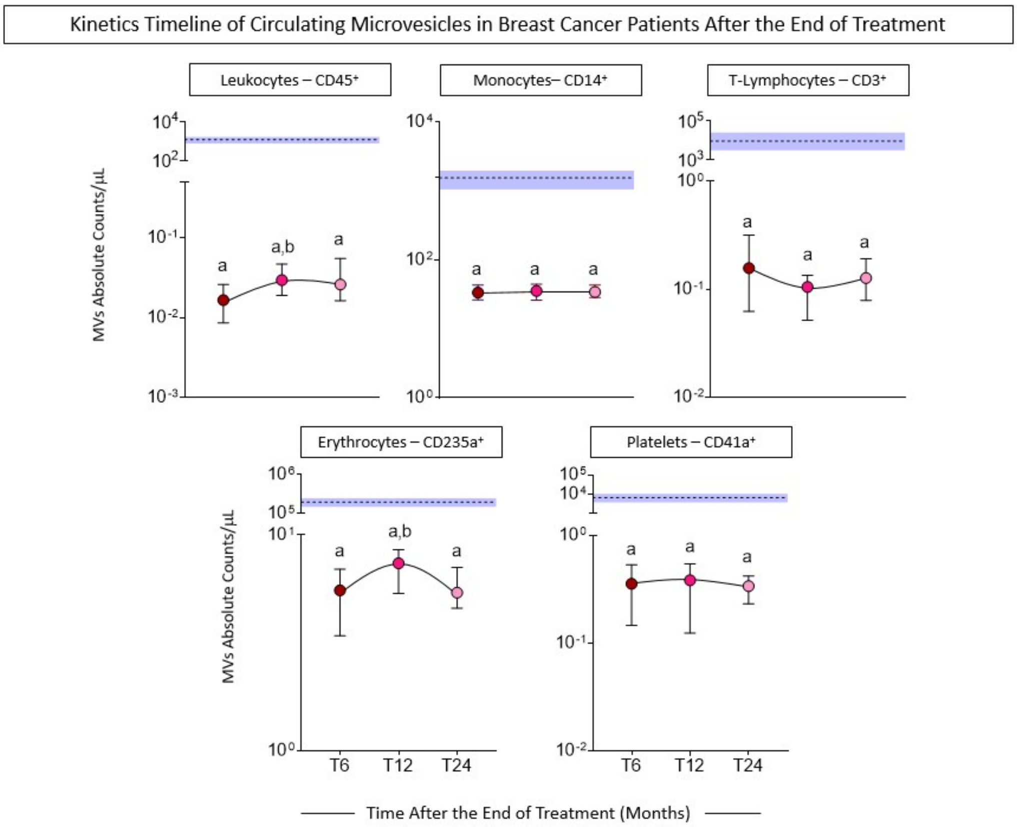

Figure S3: Quantification of circulating MVs in BC patients after six, 12 and 24 months after the end of chemotherapy and radiotherapy. Dotted line representing the median value of the total number of MVs in each group and blue band representing the interquartile ranges of the pre-treatment group (n=100). The significant differences of times T6, T12 and T24 in relation to T0 are represented by the letter "a". T6: six months pos-treatment (n = 11), T12: 12 months pos-treatment (n = 13), T24: 24 months pos-treatment (n = 11).
